# Phylogenetic Characteristics of West Nile Virus Isolated From *Culex modestus* Mosquitoes in West Kazakhstan

**DOI:** 10.3389/fpubh.2020.575187

**Published:** 2021-02-12

**Authors:** Talgat Nurmakhanov, Yerlan Sansyzbaev, Boris Atshabar, Vladimir Berlin, Damir Kobzhasarov, Olzhas Yeskhojayev, Anna Vilkova, Timur Ayazbayev, Alexey Andryuchshenko, Fyodor Bidashko, John Hay, Alexandr Shvetsov

**Affiliations:** ^1^M. Aikimbayev's Kazakh Scientific Centre for Quarantine and Zoonotic Diseases (KSCQZD), Almaty, Kazakhstan; ^2^Institute of Microbiology and Immunology, Ministry of Education and Science, Almaty, Kazakhstan; ^3^Committee on Consumer Rights Protection, Nursultan, Kazakhstan; ^4^Ural Anti-Plague Station, Uralsk, Kazakhstan; ^5^Department of Microbiology and Immunology, Jacobs School of Medicine, Univertsity at Buffalo, Buffalo, NY, United States; ^6^National Center for Biotechnology, Nursultan, Kazakhstan

**Keywords:** E segment, phylogenetic analysis, West Kazakhstan region, seroconversion, West Nile virus, West Nile virus (WNV)

## Abstract

West Nile virus is widespread in southern Russia, where the fever appears annually. Since Western Kazakhstan borders on southern Russia, we examined mosquitoes in this region for the presence of West Nile virus. Virus was detected in a small proportion of *Culex modestus* mosquitoes (3/239 pools) and isolates are related to strains from Volgograd, Russia. A screen for West Nile virus IgG was conducted and ~5% of the local human population tested positive.

## Introduction

West Nile Virus (WNV) is a virus of arthropods that is classified within the *Flaviviridae* family. It is a member of the Japanese encephalitis virus (JE) virus group, whose members include Japanese encephalitis virus, Saint Louis encephalitis virus (SLE) and Murray Valley encephalitis virus (MVE) (1). WNV is widespread in Africa, Asia, Europe, and Australia and has been responsible for several significant epidemics [Israel (1950), France (1962), South Africa (1974) and Romania (1996)] (2–5). In 1999, WNV caused two epidemics. One occurred in Volgograd, Russia, and another in the New York area, USA, where there were 62 confirmed human cases, with six deaths (2, 6–8). WNV continues to be a rising health care problem in the twenty-first century. In 2018, for example, southern and southeastern Europe saw a large increase in cases; a plausible cause for this is climate change, with early Spring and a hot Summer leading to increased mosquito activity (9–11).

West Nile fever has been diagnosed in 16 regions of the Russian Federation (12), several bordering on NW Kazakhstan. No West Nile cases have been reported (or looked for) in Kazakhstan but, given the situation in these neighboring areas of Russia where cases of WNV have been reported (Volgograd, Astrakhan, Omsk, Samara, Chelyabinsk, Orenburg) (13, 14) there is cause for concern. We therefore conducted a study of local mosquitoes in West Kazakhstan oblast as possible carriers of WNV. The main *objectives* of our study were to determine: (1) whether WNV circulated in west Kazakhstan and (2) if so, what were the dominant species of mosquito involved in the region. We employed RT-PCR for viral RNA, followed by partial sequencing and phylogenetic analyses of isolates to determine how Kazakh strains may be related to strains from Russia and other parts of the world.

## Methods

Mosquitoes for the study were collected using human landing catch approaches on collecting scientists, usually with a Mondchadskiy Bell (a 2 × 1.4 m calico cone held above the collector, acting as bait). Collections took place on 28 different occasions in all 11 districts of WKO in the area between 49.0401 and 51.2520 degrees North and 47.3325 and 54.0851 degrees East between May 25 and July 09, 2015. This area is ~100 km south of Oral, Kazakhstan and ~600 km east of Volgograd, Russia. Supplementary Figure 1 shows the geographic location of the collecting area inside a black-bordered oval at the top left-hand corner of the map. Each mosquito was identified visually by qualified, experienced entomologists using Kazakh national identification criteria. We did not tabulate males vs. females but, given the collection method, a substantial number is likely to be female. Individual insects were pooled with 35–40 copies of the same species; these are standard numbers for official collections in this part of Kazakhstan. These were stored frozen (−70°C) as whole insects. To each frozen mosquito pool, 700 μl of PBS was added prior to immediate treatment in MK28R tissue homogenizing tubes using a Bead Beater homogenizer (Retsch MM400). To isolate viral RNA, 100 μl of the suspension was used with the “AmpliPraym Ribo-Sorb” kit (Russia). The first step in the process involves isothiocyanate denaturation/stabilization.

To detect viral RNA in mosquito pools, qRT-PCR analysis using viral E (envelope) gene sequences was carried out with a hybridization-fluorescent detection method, using the “AmpliSens WNV-FL variant FRT” kit (Russia). A Rotogene 6000 cycler was employed. This is a “single-tube” method, whereby RNA to cDNA to PCR product is carried out all in the same reaction mix.

Two samples showing Ct scores of 26–28 were chosen for sequence analysis. The third positive sample had a Ct score of 36 and was not used.

For sequence analysis, the above two samples were then subjected to further RT-PCR to generate a ~900b fragment from the E gene prior to sequencing. We designed primers for nested PCR with the BioEdit Sequencing Alignment Editor (http://www.mbio.ncsu.edu/bioedit/bioedit.html) using 70 published genome sequences of WNV. Targeting conservative areas of the viral E gene with minimal nucleotide polymorphism, we selected four degenerate primers as follows:

WNV f1 1330 GGACTGTTTGGRAARGGAAGCATTGA;

WNV f2 1400 CATCCAGAARGAGAAYATCAAGTA;

WNV r1 2444 ACGGGCATTGATKCCCATCCACA;

WNV r2 2305 AACTGAYCCAAARTCCCAAGC.

These nested primers were then used in a standard two-step amplification procedure, using HotStarTaq Plus DNA polymerase (QIAGEN), as follows:

**Master Mix:**

**Table:** 

**Component name**		**Final conc**	**First round of PCR (μl) (*n* = 1)**	**Second round of PCR (μl) (*n* = 1)**
PCR Buffer with 15 mM MgCl2	10x	1x	3	3
Q-Solution	5x	1x	6	6
MgCl2	25 mM	1 mM	1.2	1.2
HotStarTaq Plus DNA Polymerase	5 u/μl	2 units/reaction	0.4	0.4
dNTP	2 mM of each	0.2 mM of each	3	3
WNV f1 1330	18 μM	600 nM	1	–
WNV r1 2444	18 μM	600 nM	1	–
WNV f2 1400	18 μM	600 nM	–	1
WNV r2 2305	18 μM	600 nM	–	1
cDNA			5	–
PCR product of the first stage			–	3
RNase-free water			9.4	11.4

**Run Parameters:**

**Table:** 

**Step name**	**First round of PCR**			**Second round of PCR**		
	**Temp (^**°**^C)**	**Time (min:s)**	**Cycles**	**Temp (^**°**^C)**	**Time (min:s)**	**Cycles**
Denature	95	05:00	1	95	05:00	1
Denature	95	0:30	35	95	0:30	35
Aneling	60	0:50		52	0:50	
Amplify	72	01:30		72	01:30	
Amplify	72	10:00	1	72	10:00	1

**Figure 1:**
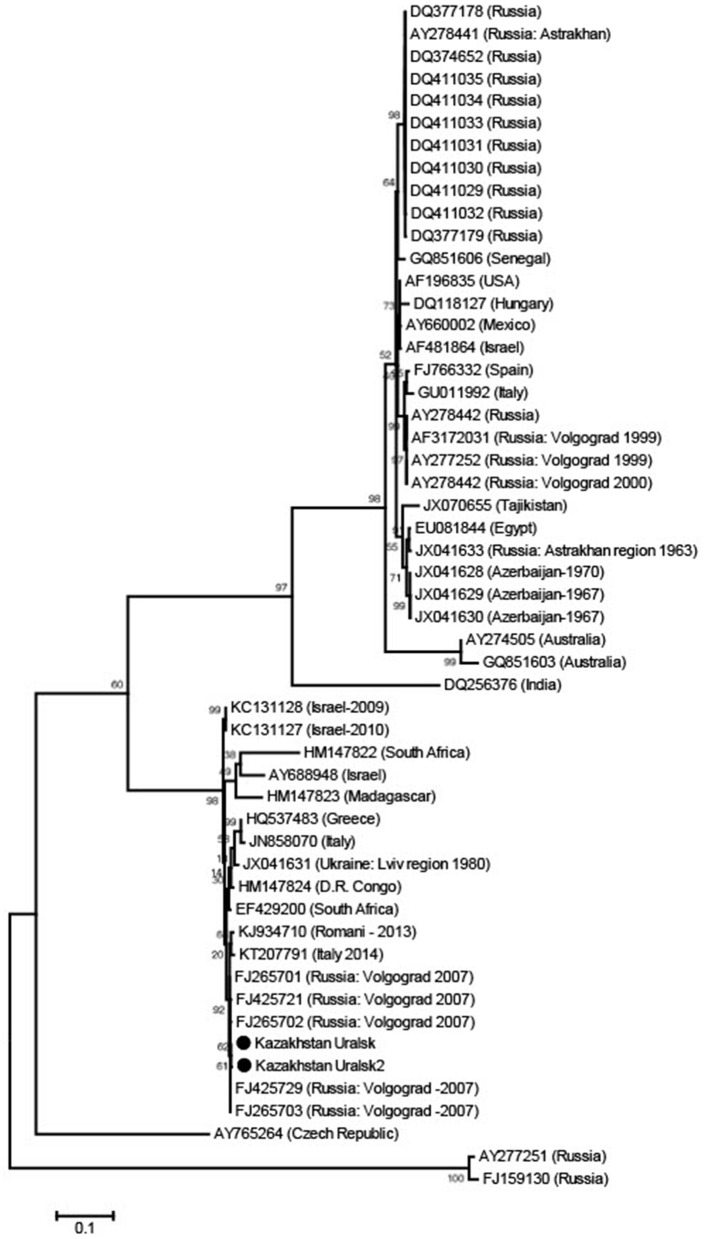
Phylogenetic tree from WNV E gene sequences using Maximum Likelihood. The sequences were obtained from *C. modestus* mosquitoes collected in NW Kazakhstan.

After checking a small sample of the reaction mix on a 1.5% agarose gel that the product was the expected ~900 base band, purification of this PCR fragment from primer residues and dNTPs was performed by precipitation in 20% PEG. Sequencing was performed using Big Dye Terminator v 3.1. (Thermo Scientific) according to the manufacturer's instructions. Sequencing reactions were performed on an automated DNA Analyzer 3730xl (Applied Biosystems). Nucleotide sequences of about 800 bases were obtained and the sequences were placed in GeneBank (GeneBank accession numbers KX129740.1; KX129741.1). Sequences obtained in two rounds of PCR were combined into a common sequence using SeqScape 2.6.0 software (AppliedBiosystems). Reference strain sequences deposited in the international NCBI database (http://www.ncbi.nlm.nih.gov/) were used as comparison. Dendrograms were constructed using Mega 6.0 software, alignment of nucleotide sequences was carried out using the Muscle algorithm and phylogenetic trees were constructed using Maximum Likelihood (Figure 1). Multiple sequence alignments, using all known sequences of the WNV E gene, were performed with Muscle in MEGA 6.0 software (15).

**The analysis details are as follows:**

Phylogeny reconstruction using Maximum Likelihood
Phylogeny Test: Bootstrap method with 1,000 replications
Substitution Model: Type-nucleotide, method—Kimura 2-parameter model
Rates/Patterns: Rates among sites—Gamma distributed; 5 discrete categories
Data subset to use gaps/missing data—complete deletion
**Tree inference options:**
  ML heuristic method—Nearest Neighbor Interchange
  Initial tree for ML—Make automatically (default—NJ/BioNJ)
  Branch swap filter—very strong
System resource usage—1st+2nd+3rd + non-coding
No. of seqs—53
No. of sites—692
No. of bootstrap reps—1,000

Following the mosquito findings, we ran a screen of 454 sera from inhabitants in the WKO, selected randomly. These serum samples had been collected by the Kazakh health authority in west Kazakhstan as part of routine screening activities and were kindly made available to us as anonymous numbered material (i.e., no identifying marks). A commercial kit was used (VectoNile-IgG, Vector Best, Russia), following the manufacturer's instructions. These kits contain positive and negative controls and have the CE seal of approval from the EU. Briefly, 1:10 and 1:100 dilutions of sera were used in 96 well plates, in duplicate, and the assays were carried out twice. Final readings were the average of these four readouts.

## Results

Mosquitoes were collected from 11 districts of the West Kazakhstan Oblast (WKO) and were identified by experienced entomologists, using Kazakh local morphology criteria. A total of 9,500 mosquitoes was combined into 239 pools. We identified insects in three genera and seven species. We did not differentiate between males and females.

The dominant potential vectors were of the genus *Culex*, representing 56.2% of the total collection, and all in one species—*Culex modestus*. The composition of mosquitoes of the genus *Aedes* was more diverse and was represented by four species—*Aedes flavescens, Aedes caspius, Aedes vexans*, and *Aedes cinereus*. Mosquitoes of the genus *Anopheles* were less common in the region, with 1.8% of the total number of captured insects, represented by two species—*Anopheles maculipennis* (we did not differentiate between sl and ss) and *Anopheles hyrcanus*. In this study, only *Culex modestus* harbored WNV. Real-time analysis by RT-PCR revealed 3 positive samples for WNV RNA in *Culex modestus* found in the Akzhaiksky area (Table 1). This area of Kazakhstan is between Oral in the north and the Caspian Sea in the south and is about 80 km from the Russian border, between Saratov and Volgograd. Comparing the Kazakh virus amino acid sequences with other strains in the NCBI database, it is clear that viruses from the WKO have a close relationship to strains from Volgograd. The high aggressiveness and anthropophilia of *Culex modestus* lend credence to the finding that it is an active carrier of WNV (16).

**Table 1:** The table provides data on the mosquito numbers captured by district, number of pools, and species of mosquito.

**Districts of the WKO**	***Culex modestus***	***Aedes flavescens***	***Aedes caspius***	***Aedes vexans***	***Aedes cinereus***	***Anopheles maculipennis***	***Anopheles hyrcanus***
Bokeyordinsky	201/4/0	–	–	–	–	–	–
Zhanibeksky	103/3/0	–	–	–	–	46/2/0	–
Zhangalinsky	1696/34/0	–	530/16/0	–	–	–	100/3/0
Kaztalovsky	12/2/0	138/5/0	118/3/0	2/1/0	–	–	–
Akzhaiksky	1080/24/3	11/1/0	259/9/0	–	–	68/5/0	74/2/0
Terektinsky	1359/28/0	–	–	387/8/0	–	50/1/0	–
Karatobinsky	376/8/0	–	20/1/0	–	19/1/0	–	–
Chingerlausky	442/11/0	1248/35/0	–	–	–	50/1/0	–
Burlinsky	–	–	–	–	–	160/4/0	–
Zelenovsky	73/2/0	–	–	–	–	–	–
Syrymsky	–	–	–	878/25/0	–	–	–
Total:	5342/116/3	1397/41/0	927/29/0	1267/34/0	19/1/0	374/13/0	174/5/0
Total (%)	56,2/48,5/ 1.2	14,7/17,2/0	9,8/12,2/0	13,4/14,2/0	0,2/0,4/0	3,9/5,4/0	1,8/2,1/0

WKO, West Kazakhstan Oblast.

*Number of mosquitoes/Number of tested pools/WN virus positive pools*.

Our IgG screen of human serum samples for WNV IgG, showed that about 5% of the samples (21 of 454) we were given were positive. Thus, while there have been no formal reports of WN fever in WKO, as stated earlier, it seems that a) the virus circulates in WKO and b) that people are being infected by it.

## Discussion

The main findings in this paper are that WNV circulates in NW Kazakhstan and is associated with *C. modestus* mosquitoes, but not in any others in our sample of positive insects.

This mosquito is a known WNV vector, is prevalent in south and southeastern Europe and seems to be moving North, perhaps in response to climate change, as mentioned earlier.

The WNV strains we identified are very similar to those in Volgograd, Russia, about 600 km away from the collecting region in Kazakhstan.

The question arises concerning the origins of positive human serum samples collected in this region and whether those were individuals who had perhaps been infected in Russia, while visiting. While this cannot be ruled out, it is unlikely that this would explain all of the cases. The area where the positive sera originated is, in fact, not directly bordering Russia (several 100 km away) and there is limited travel between it and NW Kazakhstan. It seems more likely that people are being infected by resident *C. modestus* mosquitoes. As a comparison, analogous reports of WNV prevalence in mosquitoes during outbreaks of disease in New York and Louisiana, USA, revealed mosquito infection rates similar to those found in this study (17, 18).

It would have been useful for us to have been able to further test the individuals who were positive for WNV IgG, perhaps by looking for virus in blood, or in urine where the virus seems to persist for considerable times in certain cases (19). This hopefully would have yielded virus sequences for comparison. However, as pointed out earlier, the serum samples to which we had access had been collected separately from this study and we had no means of accessing these individuals for further testing. It will be important to build on these preliminary human sample findings and we plan additional work on IgM, as well as possible serum and urine analyses for the presence of WNV genomes.

Detection of WNV in *Culex modestus* in the northwest of Kazakhstan points to a focus of infection in this region. The public health authorities in the Republic will now hopefully mount careful monitoring of the mosquito population (particularly *C. modestus*) in WKO as well screening the population for signs of West Nile fever. Several other areas of Kazakhstan border with areas of Russia with known WNV cases, many relatively unpopulated, but some with cities (e.g., Aktobe in the North and Atyrau in the West) where continued exploratory work on WNV in Kazakhstan would be an important future public health activity.

## Data Availability Statement

The datasets presented in this study can be found in online repositories. The names of the repository/repositories and accession number(s) can be found in the article/Supplementary Material.

## Ethics Statement

The studies involving human participants were reviewed and approved by Republic of Kazakhstan. Written informed consent for participation was not required for this study in accordance with the national legislation and the institutional requirements.

## Author Contributions

All authors listed have made a substantial, direct and intellectual contribution to the work, and approved it for publication.

## Conflict of Interest

The authors declare that the research was conducted in the absence of any commercial or financial relationships that could be construed as a potential conflict of interest.
