# Supplementary material for: Phylogenetic Characteristics of West Nile Virus Isolated From Culex modestus Mosquitoes in West Kazakhstan
Source: Front Public Health. 2021 Feb 12;8:575187. doi: 10.3389/fpubh.2020.575187 (PMC7907182; doi:10.3389/fpubh.2020.575187)
Supplement: Supplementary file 1 [file Image_1.pdf]

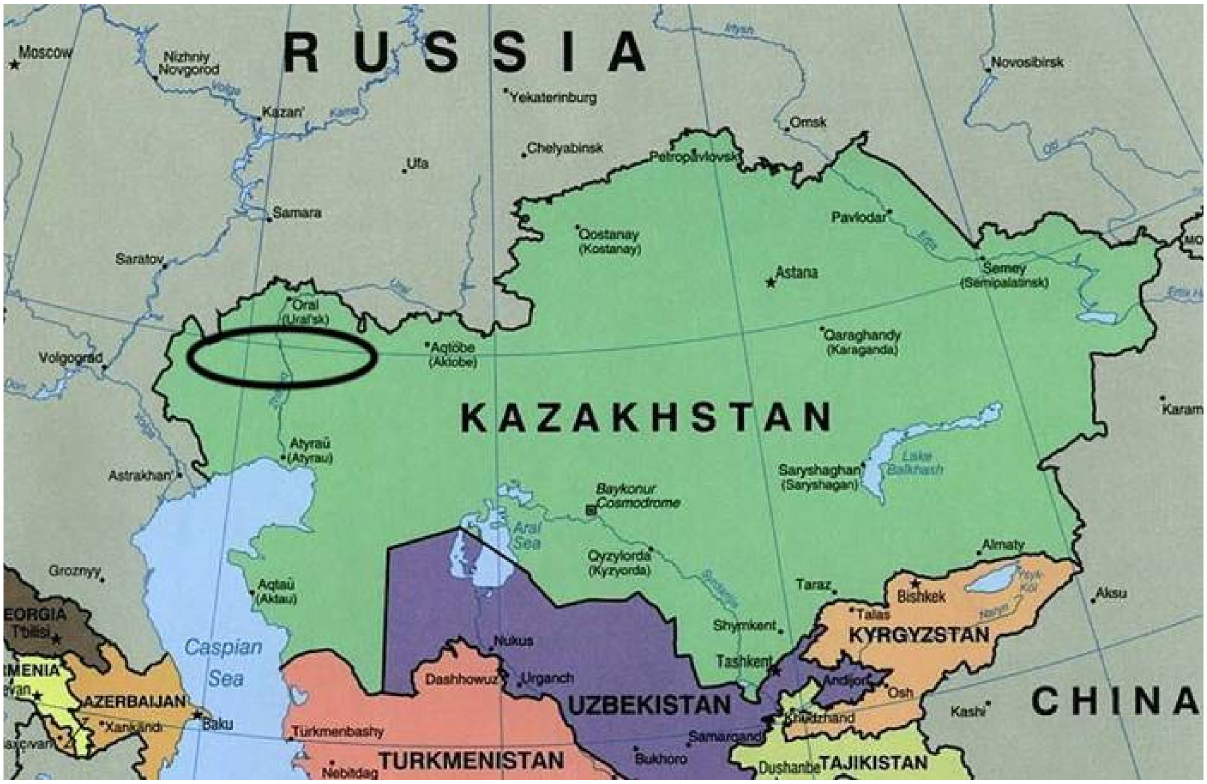

Figure 1: The location of mosquito and human blood collections in NW Kazakhstan is shown by the black-bordered oval in the top left-hand area of the map. Credit: CIA - Caucasus and Central Asia.
